# Supplementary material for: MicroRNA Expression in Extracellular Vesicles from Nasal Lavage Fluid in Chronic Rhinosinusitis
Source: Biomedicines. 2021 Apr 26;9(5):471. doi: 10.3390/biomedicines9050471 (PMC8145239; doi:10.3390/biomedicines9050471)
Supplement: Supplementary file 1 [file biomedicines-09-00471-s001.zip › biomedicines-1191832-Supplementary.pdf]

biomedicines

Supplementary

Table S1. Demographics of CRS patients and healthy subjects.

	CRSwNP (*N = 44*)	CRSsNP (*N = 108*)	HC (*N = 77*)
Sex, male (%)	27 (61.36%)	75 (69.44%)	47 (61.04%)
Age, year	45 (2.6)	44 (1.8)	37 (2.4)
JESREC score	10 (0.7)	6 (0.4)	1 (0.7)
**Eosinophil**			
non-ECRS	25 (55.56%)	86 (86.00%)	11 (100.00%)
mild ECRS	12 (26.67%)	10 (10.00%)	0 (.00%)
moderate ECRS	4 (8.89%)	1 (1.00%)	0 (.00%)
severe ECRS	4 (8.89%)	3 (3.00%)	0 (.00%)
**Allergy**			
(+)	11 (25.58%)	44 (41.90%)	6 (7.79%)
(-)	32 (74.42%)	61 (58.10%)	71 (92.21%)
**Asthma**			
(+)	5 (11.11%)	4 (3.74%)	2 (2.60%)
(-)	40 (88.89%)	103 (96.26%)	75 (97.40%)

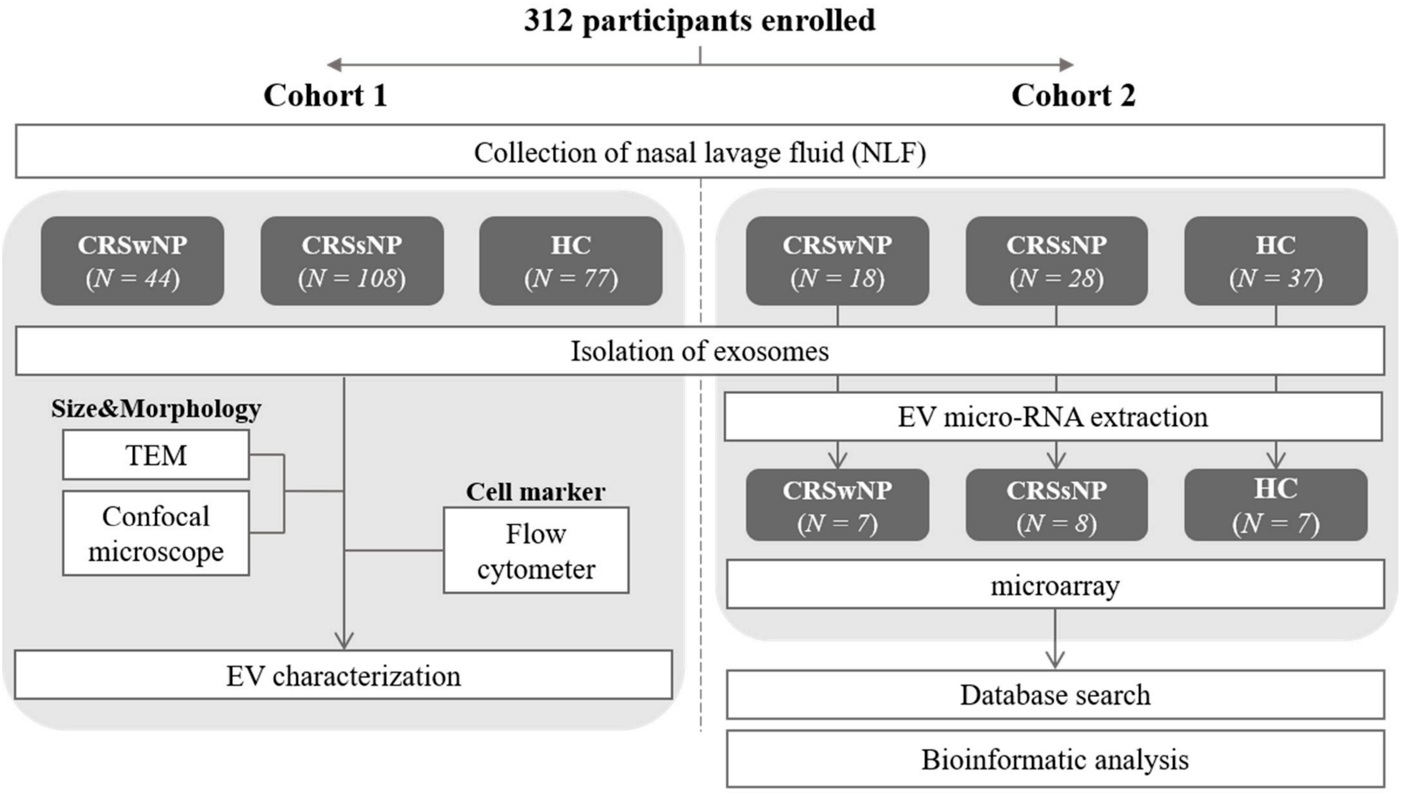

Figure S1. Study cohorts and participation: scheme of approach to include and analyze the study population.

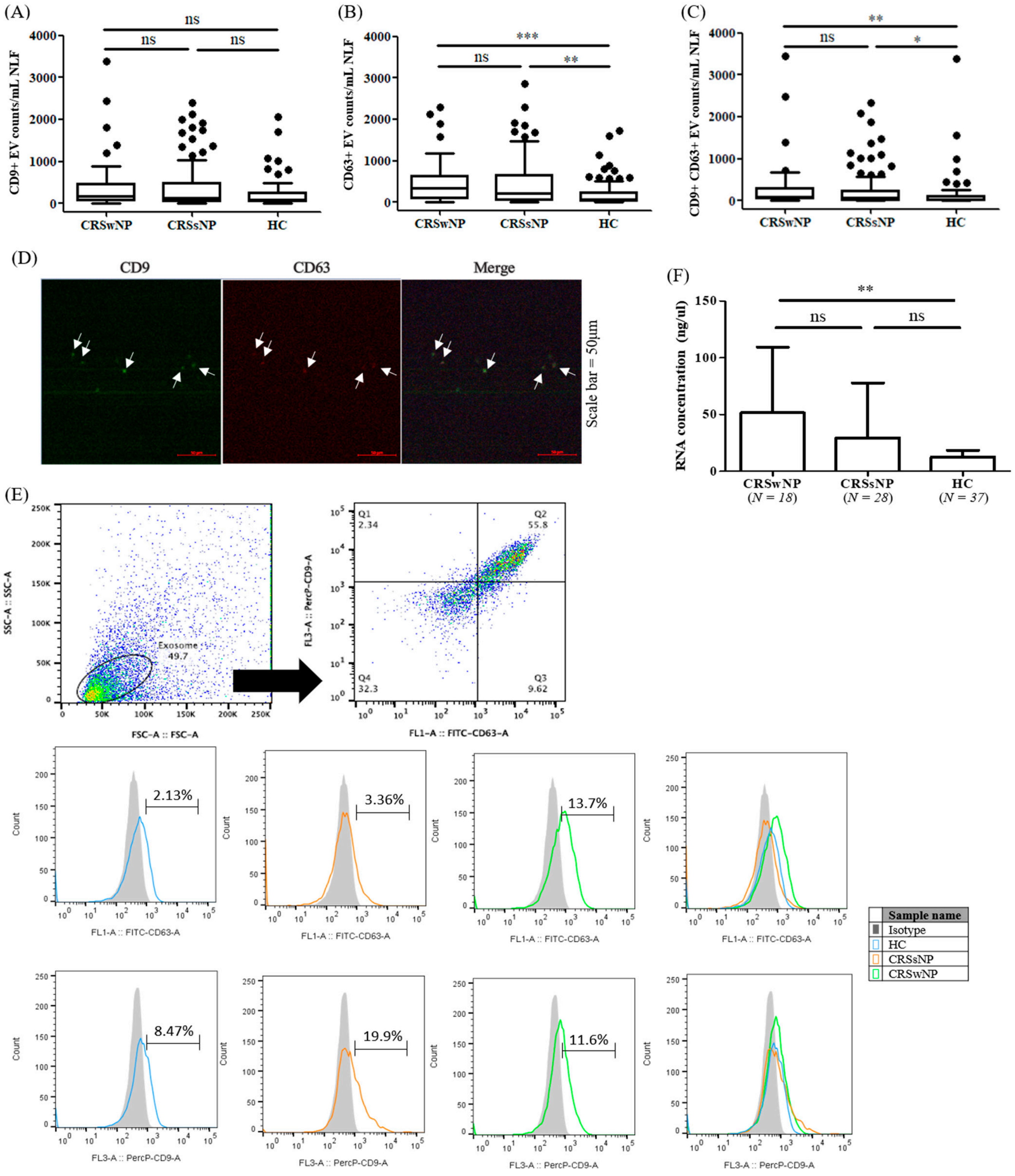

Figure S2. Phenotypic characterization of NLF EVs and concentration of total RNA in EVs. Phenotypic markers were analyzed by FCM, and distributions of (**A**) CD9+ EVs, (**B**) CD63+ EVs and (**C**) CD9+/CD63+ EVs were shown by patient group. (**D**) EVs were visualized using FITC-CD9 and PercP-CD63 markers by confocal microscopy and (**E**) FCM analysis of EVs displayed expression of FITC-CD9 and PercP-CD63 markers. (F) Total RNA concentration (ng/μL) in NLF-EVs by groups. 1-way ANOVA was used to evaluate statistical differences between groups. Levels of statistical significance are expressed as * $p < 0.05$, ** $p < 0.01$, *** $p < 0.005$ and ns > 0.05.
